# An Unusual Cause of Hexokinase 1 Deficiency—Case Report

**DOI:** 10.1002/jha2.70123

**Published:** 2025-08-23

**Authors:** Gonench Kilich, Kelly Maurer, Tanaya Jadhav, Tejas Jammihal, Zhe Zhang, Helge Hartung, Kosuki Izumi, Kelly Hassey, Anna Raper, Erica Schindewolf, Laura Conlin, Rebecca Ganetzky, Marilyn Li, Bertil Glader, Ramakrishnan Rajagopalan, Kathleen E. Sullivan

**Affiliations:** ^1^ Division of Allergy Immunology Children's Hospital of Philadelphia Philadelphia Pennsylvania USA; ^2^ Division of Genomic Diagnostics Department of Pathology and Laboratory Medicine Children's Hospital of Philadelphia Philadelphia Pennsylvania USA; ^3^ Department of Computational Biology St. Jude Children's Research Hospital Memphis Tennessee USA; ^4^ Clinic for Special Children Intercourse Pennsylvania USA; ^5^ Department of Pediatrics Children's Medical Center Research Institute at UT Southwestern Dallas Texas USA; ^6^ Division of Translational Medicine and Human Genetics Perelman School of Medicine Philadelphia Pennsylvania USA; ^7^ Division of Human Genetics Children's Hospital of Philadelphia Philadelphia Pennsylvania USA; ^8^ Pediatric Hematology/Oncology, Department of Pathology Bass Cancer Center For Childhood Cancer and Blood Diseases Stanford, Palo Alto California USA

**Keywords:** case report, hereditary hemolytic anemia, non‐coding mutation

## Abstract

**Introduction:**

Molecular analysis of red cell disorders has revolutionized diagnosis, however, there remain challenges.

**Main Symptoms:**

This patient presented with hemolytic anemia in the newborn period. He required chronic transfusions to maintain his hemoglobin level until 6 years of age. A splenectomy was performed at 3 years of age.

**Main Diagnoses:**

Using whole genome sequencing, we were able to identify a duplication upstream of the red cell promoter of *HK1*. Long‐read RNA sequencing established aberrant expression off of this promoter.

**Conclusions:**

These non‐coding variants remain challenging to identify. His promoter duplication may have a founder effect in South Asia.

## 1 Introduction

**FIGURE 1:**
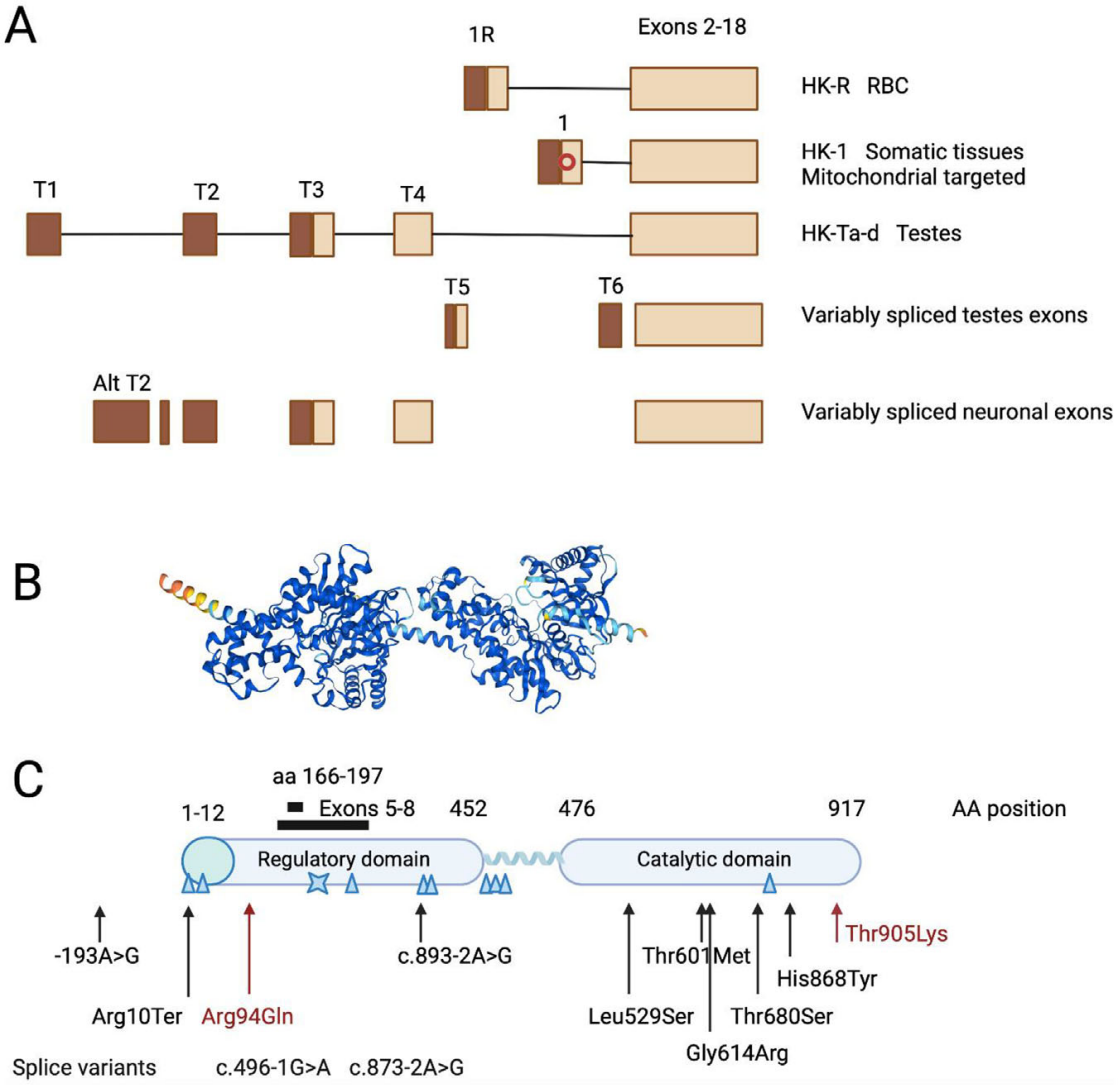
Hexokinase 1 gene, protein, and variants. (A) The alternative upstream exon utilization in different tissues is displayed. Exons 2–18 are invariant. Exon 1 is used by almost all somatic tissues and incorporates a mitochondrial targeting peptide (red circle) and the resulting transcript is termed HK1. Exon 1R is utilized in early reticulocytes and lacks the mitochondrial targeting sequence, resulting in a distinct transcript termed HK‐R. Multiple upstream exons are variably spliced in testes and neuronal tissues. Light brown represents sections that are translated while dark brown represents non‐translated components. Nomenclature of exons is according to Andreoni [5]. (B) The protein structure was exported from the Human Protein Atlas and corresponds to crystallographic data from Mulichak [21]. The N‐terminus is on the left with the first 12 amino acids of the mitochondrial‐targeting peptide shown in yellow and red. The linker that separates the regulatory domain from the catalytic domain at the C‐terminus is visible as an alpha helix in the middle. (C) Schematic representation of variants found in patients with hemolytic anemia with the variants listed at individual locations. The blue circle defines the porin‐binding domain. The variants with locations defined in dark red represent those described with a concomitant neurologic phenotype. The patient with Arg94Gln had a CNS bleed at birth, which might have led to developmental delay [11]. The patient with Thr905Lys had microcephaly and developmental delay but was diagnosed at 3 months of age. The blue triangle symbols correspond to the autosomal dominant variants found in various neurologic conditions. Triangles represent missense variants and the blue X represents a termination event. These variants are displayed only schematically. Variants were taken from ClinVar and Okur [13].

Hexokinase (HK) converts glucose to glucose‐6‐phosphate in the glycolytic pathway [1]. *HK1* encodes multiple tissue‐specific isoforms and *HK‐R* expression is driven by an erythroid‐specific promoter, active in very early erythroid development [2]. HK‐R lacks the porin domain at the N‐terminal, which targets HK1 to the mitochondria (Figure 1) [1, 2, 3, 4]. Testes and neurons utilize alternative upstream exons but most tissues express the canonical HK1 isoform [5].

Autosomal recessive (AR) HK1 deficiency causes hereditary hemolytic anemia with neonatal or fetal presentation (Figure 1C) [6, 7, 8, 9, 10, 11, 12]. In contrast, autosomal dominant HK1 deficiency is associated with neurologic conditions [8, 13, 14, 15, 16, 17] and large deletions of non‐coding DNA at this locus have been associated with congenital hyperinsulinemia [18] (Figure 1C). The non‐hematologic conditions are generally due to distinct mutations, although hemolysis combined with a neurologic phenotype has been described (Figure 1C, variants in red font) [13]. Fewer than 30 cases of AR HK1 deficiency have been described with only half having molecular characterization. Variants causing isolated hemolytic anemia are typically in the catalytic domain (Figure 1C) while variants associated with neurologic phenotypes are typically in the regulatory domain (Figure 1C, blue symbols).

## 2 Patient Information

We report a boy of South Asian descent who was transfusion dependent since three months of age. He was born to parents who are first cousins. He had anemia (hematocrit 25%) and thrombocytopenia (platelet count 49,000/µL) noted at birth. An infection workup was negative, and there was no blood group incompatibility. His hemoglobin electrophoresis was normal and metabolic testing was unrevealing.

## 3 Clinical Findings and Timeline

At 3 weeks of age, his hemoglobin was 6.2 g/dL, MCV 126 fL, platelets 52,000/µL, with 9% band cells, 4% metamyelocytes, 1% blasts, and elevated absolute neutrophils at 12,640/µL. A bone marrow biopsy showed mildly hypercellular marrow (100%), and absolute erythroid hyperplasia. He was started on monthly transfusion therapy at 3 months of age and at 3 years of age, he underwent splenectomy for hypersplenism.

He had normal growth and development as defined by meeting developmental milestones. On transfusion therapy, he had reticulocytes of 15%–20% and hemoglobin of 9–9.5 g/dL until about 6 years of age (2022) at which point his dependency on transfusions resolved. Stem cell transplantation was discussed, but a lack of diagnosis led him to the Undiagnosed Diseases Network.

## 4 Diagnostic Testing

His diagnostic journey included karyotype, whole‐exome sequencing, mitochondrial testing, and anemia/cholestasis panels, which were non‐diagnostic. A chromosomal microarray was sent, which was notable for 150 kb duplication on chromosome 13q34 and areas of homozygosity totaling 3%, including the *HK1* gene.

His phenotype most closely resembled an enzymopathy. Centrifuged blood enriched for older RBCs in the bottom layer (transfused cells) and the patient's own reticulocytes in the top layer. The patient had equivalent levels of HK in both layers, whereas typically younger cells would have higher levels of all enzymes (Table S1). RBCs express both HK‐R and HK1 with HK‐R protein highest in the earliest reticulocytes and is absent in peripheral blood. HK1, in contrast, declines steadily throughout the lifespan of the RBC [3, 7].

**FIGURE 2:**
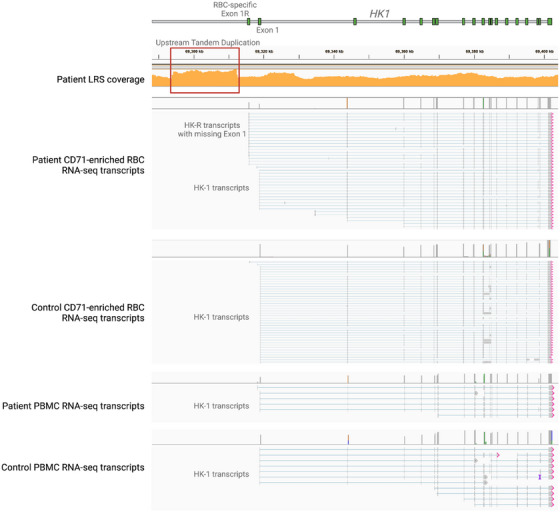
HK1 promoter duplication impacting RBC transcription. The upstream tandem duplication is displayed in the red box in the top LRS coverage tracing. CD71 reticulocyte‐enriched RBC long‐read RNA‐seq tracings demonstrate two main transcript types in the patient with most originating from the RBC‐specific exon 1 and others originating from the canonical HK‐1 exon 1. The control long‐read RNA‐seq reads are from a sex‐matched healthy donor and show the expected dominance of the canonical HK1 transcripts for peripheral blood reticulocytes. The PBMC transcripts are comparable between patient and control and arise from the canonical HK1 exon 1.

Standard analysis of whole genome sequencing was not revealed initially. Manual review for CNV impacting regulatory regions revealed a homozygous duplication (GRCh38 chr10:69,293,869–69,312,244×4) upstream of the RBC‐specific exon (Figure 2). This duplication was identified by the CNV caller but was not recognized as clinically relevant. De novo assembly of long‐read DNA sequencing identified the tandem duplication on both haplotypes. Altered RBC‐specific transcripts were identified using long‐read RNA‐seq on patient and control reticulocytes enriched using CD71 beads/streptavidin. The patient exhibited persistence of the HK‐R transcripts, which lack a mitochondrial targeting domain. Lack of mitochondrial targeting has been associated with increased apoptosis, suggesting a mechanism of disease [19]. PBMC transcripts used the canonical *HK1* exon 1, comparable to control (Figure 2). Therefore, this promoter tandem duplication led to persistence of the HK‐R isoform without altering the canonical HK1 isoform.

## 5 Therapeutic Intervention and Outcomes

Management of HK deficiency is variable with some patients spontaneously improving and some responding to splenectomy. Hematopoietic stem cell transplant has been performed for other red cell enzymopathies [20]. For this patient, the decision to pursue transplant was deferred due to uncertainty of diagnosis. In the interim, his transfusion dependency resolved.

## 6 Discussion

Previously, a promoter variant affecting AP‐1 binding upstream of the *HK‐R*‐specific exon was described as a cause of hemolytic anemia [7]. Non‐coding variants remain difficult to prioritize using standard bioinformatic analysis as the workflows are biased for protein‐coding regions and the duplication in our patient was too small to be detected on an SNP array. CNV reporting criteria vary according to laboratories and technology. Duplications are generally reported when they are >200 kb or span at least 10 probes on SNP array. In contrast, genome‐based CNV detection relies on binned read depth, and prioritizes based on content and potential impact of the CNV rather than its size alone. Additionally, computational tools for prioritizing noncoding variants remain limited. This case highlights the need for routine clinical workflows to incorporate features to prioritize non‐coding variants.

This duplication impacted expression of the RBC‐specific transcripts with persistence of this isoform. Two very similar duplications have been reported in GnomAD in the heterozygous state in eight individuals, six of whom were of South Asian ancestry, suggesting a possible founder effect. For AR conditions, carrier screening is advised for variants with a frequency of ≥1 in 200, while conditions with a frequency of ≥1 in 100 may be considered for newborn screening (ACMG). This variant has a frequency of 0.6%, supporting its inclusion in carrier screening.

Long‐read DNA sequencing enabled phasing of the homozygous duplications, while long‐read RNA sequencing provided precise quantification of isoforms, together facilitating comprehensive molecular resolution of the variant's impact. Although long‐read sequencing is gaining traction in both research and select clinical applications, its broader adoption remains limited due to lower throughput and higher costs.

In summary, establishing a genetic etiology required a comprehensive approach, including detailed phenotypic assessment, advanced enzymatic analysis, detection of loss of heterozygosity at the HK1 locus, and long‐read RNA sequencing. Fortunately, the patient has done well.

## Author Contributions

K.M. performed wet bench studies. G.K., K.E.S., R.G., E.S., A.R., K.H., K.I., H.H. all worked to collect key clinical details and analyzed data returned from investigations. Z.Z., Ta.J., Te.J., R.R., M.L., and L.C. all performed advanced analyses of the DNA and RNA sequencing. B.G. performed enzyme testing.

## Ethics Statement

This study was approved as a Single IRB at NIH and as a relying site by Children's Hospital of Philadelphia.

## Consent

The patient's family provided informed consent for testing and publication.

## Conflicts of Interest

The authors declare no conflicts of interest.

## Supporting information




**Supplemental Table 1**: Direct enzyme analysis on reticulocyte enriched cells.

## Data Availability

Primary data are available upon request to K. E. Sullivan.
