# Supplementary material for: An Unusual Cause of Hexokinase 1 Deficiency—Case Report
Source: EJHaem. 2025 Aug 23;6(4):e70123. doi: 10.1002/jha2.70123 (PMC12374552; doi:10.1002/jha2.70123)
Supplement: Supplementary file 1 — Supplemental Table 1: Direct enzyme analysis on reticulocyte enriched cells. [file JHA2-6-e70123-s001.docx]

**Supplemental Data**

**Supplemental Table 1**

**Direct enzyme analysis on reticulocyte enriched cells**

|  | **Top (reticulocyte enriched)** | **Bottom** |
| --- | --- | --- |
| G6PD | 9.6 | 4.3 |
| 6-Phosphogluconase dehydrogenase | 4.5 | 3.3 |
| Pyruvate kinase | 10.1 | 2.9 |
| Glucose phosphate isomerase | 25.9 | 18.8 |
| Adenosine deaminase | 0.63 | 0.48 |
|  |  |  |
| Hexokinase | 0.18 | 0.18 |
